# HO-1 Induction in Cancer Progression: A Matter of Cell Adaptation

**DOI:** 10.3390/antiox6020029

**Published:** 2017-05-05

**Authors:** Mariapaola Nitti, Sabrina Piras, Umberto M. Marinari, Lorenzo Moretta, Maria A. Pronzato, Anna Lisa Furfaro

**Affiliations:** 1 Department of Experimental Medicine, University of Genoa, Via L. B. Alberti 2, Genoa 16132, Italy; Mariapaola.Nitti@unige.it (M.N.); piras.sabri@tiscali.it (S.P.); umm@unige.it (U.M.M.); maidep@unige.it (M.A.P.); 2 Bambino Gesù Children’s Hospital, IRCCS, Piazza S. Onofrio 4, Rome 00165, Italy; Lorenzo.Moretta@opbg.net; 3 Giannina Gaslini Institute, IRCCS, Via Gerolamo Gaslini 5, Genoa 16147, Italy

**Keywords:** HO-1, Nrf2, cancer progression, tumor microenvironment, immune-escape, oxidative stress, NK, melanoma, NSCLC, prostate cancer

## Abstract

The upregulation of heme oxygenase-1 (HO-1) is one of the most important mechanisms of cell adaptation to stress. Indeed, the redox sensitive transcription factor Nrf2 is the pivotal regulator of HO-1 induction. Through the antioxidant, antiapoptotic, and antinflammatory properties of its metabolic products, HO-1 plays a key role in healthy cells in maintaining redox homeostasis and in preventing carcinogenesis. Nevertheless, several lines of evidence have highlighted the role of HO-1 in cancer progression and its expression correlates with tumor growth, aggressiveness, metastatic and angiogenetic potential, resistance to therapy, tumor escape, and poor prognosis, even though a tumor- and tissue-specific activity has been observed. In this review, we summarize the current literature regarding the pro-tumorigenic role of HO-1 dependent tumor progression as a promising target in anticancer strategy.

## 1. Introduction

Heme oxygenase-1 (HO-1) is one of the three isoforms of heme oxygenase, the first rate-limiting enzyme in the degradation of heme to free iron, carbon monoxide (CO), and biliverdin [1,2]. HO-1 is a 32-kDa inducible protein identified in 1990 as a stress protein involved in antioxidant and anti-inflammatory responses [3]. HO-1 is present at low levels in most mammalian tissues and is highly upregulated by a number of oxidative stimuli such as its substrate heme, heavy metals, UV irradiation, ROS, modified lipids, growth factor, and inflammatory cytokines [3,4,5]. HO-1 is mainly localized in microsomes [6], but it has also been demonstrated to be present in caveolae [7], mitochondria [8], and nuclei [9]. 

**Figure 1:**
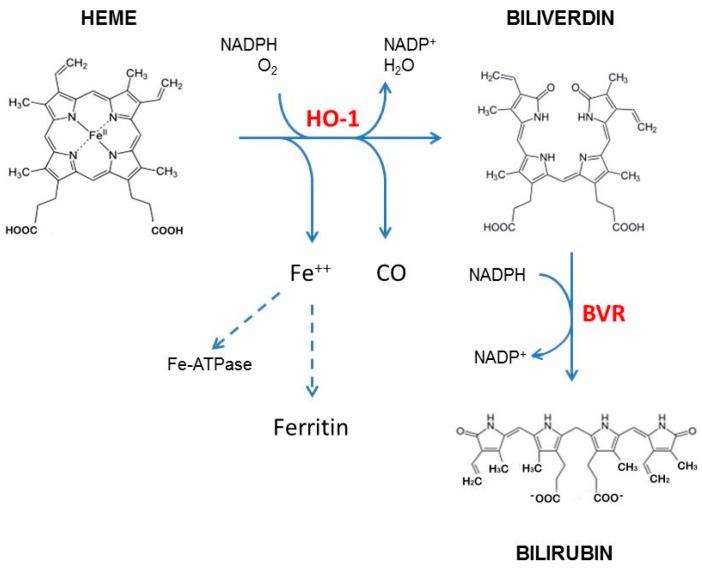
Heme degradation pathway. Heme oxygenase-1 (HO-1) catalyzes the degradation of heme to equimolar amounts of carbon monoxide (CO), biliverdin, and ferrous iron in presence of molecular oxygen (O_2_) and nicotinamide adenine dinucleotide phosphate (NADPH). Biliverdin is subsequently converted to bilirubin by biliverdin reductase (BVR).

The cytoprotective activity of HO-1 is exerted by its metabolites [2,10] (Figure 1). Indeed, the release of free iron favors the synthesis of the heavy chain of ferritin, an iron chelating protein, and the activation of the membrane transporter Fe-ATPase, which permits cytosolic iron efflux, decreasing the intracellular Fe^2+^ content preventing the generation of ROS through the Fenton reaction [11,12]. Furthermore, carbon monoxide exerts antiapoptotic and anti-inflammatory effects through the induction of soluble guanylyl cyclase (sGC), elevation of cGMP, and modulation of mitogen-activated protein kinase pathway (MAPK) [2,10,13]. As a consequence, HO-1–derived CO stimulates blood vessel formation [14], induces VEGF synthesis [15], and favors the proliferation of endothelial cells [16] crucially involved in wound healing [17]. In addition, bilirubin (BR) derived from biliverdin (BV) by biliverdin reductase (BVR), exerts strong antioxidant [18], antiapoptotic [10], and anti-inflammatory activity [19]. In fact, bilirubin is able to scavenge hydroxyl radical, singlet oxygen, and superoxide anions [20] and prevents protein and lipid peroxidation [21,22]. Moreover, bilirubin exerts anti-inflammatory effects decreasing P- and E-selectin expression, preventing leucocyte rolling and inhibiting the complement cascade [6]. Recent studies have also demonstrated that HO-1 plays an important role in immune regulation promoting immune tolerance [23].

Thus, the biological properties of HO-1 have been mainly ascribed to its enzymatic activity. However, HO-1 can also act independently of its enzymatic function. Indeed, it has been shown that HO-1 can move into the nucleus and regulate gene transcription, specifically favoring cancer tumor growth [9,24]. 

HO-1 is highly induced in oxidative stress conditions. Its synthesis is regulated mainly at a transcriptional level [13,25]. In the promoter region of HO-1, in fact, several binding sites are present for different transcription factors that can be activated in oxidative stress conditions like AP-1, HIF-1, NF-kB, and Nrf2 [26]. Among these, Nrf2 is considered a key regulator of HO-1 transcription [13]. 

The nuclear factor erythroid 2 p45-related factor 2 (Nrf2) plays a pivotal role in maintaining cellular redox homeostasis. It belongs to the Cap ‘n’ Collar (Cnc)-bZIP (basic leucine zipper) family of transcription factors together with Nrf1 and Nrf3 [27] as well as the transcriptional repressors Bach1 and Bach2 [28]. Nrf2 protein has a seven-domain structure which accounts for its binding to repressors and to DNA [29,30,31]. The binding with the E3 ubiquitin ligase Kelch ECH-associating protein 1 (Keap1) provides its constant proteasomal degradation in resting condition. When Keap1 is modified by oxidative or electrophilic stressors, Nrf2 becomes free to move into the nucleus, where it dimerizes with Maf proteins and binds antioxidant/electrophile-response elements (ARE/EpRE sequences) [31,32,33] leading to the transcription of target genes. Among these, a plethora of antioxidant and detoxifying enzymes involved in cell resistance to stress have been identified as well as regulators of cell proliferation and differentiation [27,34,35,36,37].

Furthermore, other mechanisms can induce Nrf2 activation. Due to its interaction with sequestosome-1 protein (p62/SQSTM1), Keap1 is sequestered in autophagosomes. As a consequence, Nrf2 ubiquitination decreases leading to its prolonged activation in response to oxidative stress [38,39]. It has been also demonstrated that, in a Keap1-independent manner, β-Transducin Repeat Containing E3 Ubiquitin Protein Ligase (β-TrCP) and Glycogen Synthase Kinase 3β (GSK-3β) can induce the ubiquitination and proteasomal degradation of Nrf2 [40]. 

Notably, in cancer cells, further mechanisms of amplification of Nrf2 signaling have been described [30]. Genetic modifications of Nrf2/Keap1, mainly gain-of-function mutations of Nrf2 and loss-of-function mutations of Keap1, lead to a constitutive activation of Nrf2 [41,42,43,44]. Moreover, epigenetic alterations in Keap1, such as Keap1/Cul3 hypermethylations, are responsible for the accumulation and aberrant activation of Nrf2 in lung [45], prostate [46], head and neck [47], colorectal [48], and ovarian cancer [49]. In addition, the Nrf2-Keap1 binding can be modified by the interaction with other proteins which increase Nrf2 stabilization [50]. For instance, p21 [51,52,53] as well as DJ-1 are able to bind Keap1, allowing Nrf2 to translocate into the nucleus [54]. Indeed, DJ-1 in cancer is often upregulated and increases the expression of detoxification enzymes providing a survival advantage [55,56,57].

It is important to note that the binding of Nrf2 to ARE sequences is highly complex and Bach1 represents another regulator of the transcription of Nrf2 target genes and of HO-1 in particular [58]. In fact, Bach1 is a heme-binding protein which, in unstressed conditions, dimerizes with Maf proteins and binds to the ARE/EpRE sequences, acting as transcriptional repressor of ARE-dependent genes. Under oxidative conditions or when the concentration of heme groups increases, a conformational change of Bach1 favors its displacement from ARE sequences and its degradation to proteasome, allowing Nrf2 to bind [58,59].

Notably, HO-1 activity is also regulated by specific compartmentalization of the enzyme. In fact, it has been identified as being active at a mitochondrial level in lung epithelial cells exposed to cigarette smoke, LPS, or hemin [60], while its localization at the plasma membrane prevents its catalytic activity. Indeed, in rat pulmonary artery endothelial cells it has been demonstrated that HO-1 activity is negatively modulated by Caveolin-1 (Cav-1) [7]. The five amino acid sequence in Cav-1 responsible for the binding to HO-1 has been identified [61] and the role of Cav-1 in the modulation of HO-1 activity has been recently proved by using a Caveolin-1 scaffolding domain (CSD) peptide able to decrease the compartmentalization of HO-1, increasing its activity in alveolar macrophages and in mice [62].

Therefore, in physiological conditions, the activation of Nrf2/HO-1 axis is involved in the maintenance of cellular homeostasis and plays a central role in adaptive response to cellular stress, representing a crucial point in cytoprotection, cell survival, and in the prevention of carcinogenesis [63,64,65]. By contrast, in cancer cells, a prolonged activation of Nrf2 and HO-1 is ambiguous and deleterious [30,31,66]. 

In this review, we focus our attention on HO-1 upregulation in cancer progression, analyzing its involvement in tumor growth, resistance to therapies, and in the gain of metastatic features. Moreover, a growing body of evidence demonstrates the involvement of HO-1 in the generation of a favorable microenvironment, promoting angiogenesis and immune-escape in many types of cancers including melanoma, brain tumors, NSCL, prostate cancer, and chronic myeloid leukemia [66,67,68,69,70] and this aspect will be also described. 

## 2. HO-1 in Cancer Growth and Resistance to Therapy

HO-1 expression correlates with cancer growth and resistance to therapy as shown in different types of tumors such as human renal cell carcinoma [71], prostate and pancreatic cancers [10,72], lymphosarcoma [73], melanoma, and hepatoma [74]. Moreover, anticancer treatments such as chemo-, radio-, and photodynamic therapy further increase HO-1 expression [75] and it has been demonstrated that pharmacological inhibitors of HO-1 are able to sensitize cancers to therapies [74,76]. 

Several studies highlighted that cancer cells with high levels of HO-1 are less sensitive to the treatment with etoposide, doxorubicin, or cisplatin [77,78,79,80]. In fact, HO-1 overexpression protects from antitumor activity of cisplatin both Hep-2 laryngeal squamous cells and HepG-2 hepatoma cells as well as MNK-4 gastric cancer cells and A549 lung cancer cells [80,81]. HO-1 inhibition or silencing enhances cytotoxicity [79,82] downregulating MMP-9 and VEGF levels and favoring apoptosis [80,81]. 

Moreover, HO-1 upregulation contributes to chemoresistance to doxorubicin or gemcitabine in urothelial cancers and in cholangiocarcinoma cell lines and its inhibition or silencing is able to increase ROS production and cytotoxicity and inhibit tumor growth in vivo [83,84,85]. In addition, siRNA HO-1 leads to the inhibition of neoplastic growth in human pancreatic cancer resistant to gemcitabine [86]. Interestingly, it has been shown that the autophagy pathway induced by the activaton of Src/STAT3/HO-1 protects several subtypes of breast cancer cells from doxorubicin-induced cytotoxicity [78]. HO-1 is also overexpressed in melanoma cell lines after treatment with proteasome inhibitor bortezomib and with arsenic trioxide [87,88] and, in glioma cells, the resistance to arsenic trioxide is reverted by HO-1 inhibition obtained using Zinc II Protoporphyrin IX (ZnPPIX), leading to an enhanced apoptosis [89]. Furthermore, we have demonstrated that Nrf2-dependent HO-1 induction prevents neuroblastoma cell death after GSH depletion or bortezomib treatment and HO-1 inhibition or silencing restore cell sensitivity [77,90,91]. 

As far as the radio- and photodynamic-therapy (PDT) is concerned, the overexpression of HO-1 is involved in human lung adenocarcinoma and in urothelial cancer resistance to irradiation treatment and its inhibition induced by ZnPPIX or SnPPIX enhances radiosensitivity [83,92,93]. In addition, it has been demonstrated that HO-1 silencing sensitizes human urothelial as well as HeLa cervical cancer cells to 5-aminolevulinic acid-based photodynamic therapy [75,94] and that HO-1 siRNA or inhibition increase the PDT cytoxicity in colon adenocarcinoma C-26 and human ovarian carcinoma MDAH2774 cells [95]. Also in melanoma cells, HO-1 inhibition increases the responsiveness to PDT therapy, leading to apoptosis [96].

HO-1 is involved in chemoresistance in solid tumors, but it seems, although not yet fully clarified, to play a role in hematopoietic malignancies as well. The drug-resistant acute myeloid leukemia (AML) cell line HL-60R is significantly less sensitive to cytarabine and daunorubicin than HL-60 cells and this correlates to HO-1 overexpression. Indeed, HO-1 downregulation significantly enhanced the sensitivity of HL-60R to chemotherapy [97]. Moreover, in the majority of AML patients, especially in those with acute monocytic leukemia and leukocytosis, HO-1 is aberrantly overexpressed. In this context, it has been demonstrated that in a mouse xenograft model of AML, HO-1 silencing extends the survival rate [98]. Furthermore, HO-1 protects AML cells from the apoptosis induced by proteasome inhibitor, bortezomib [99], or to front-line chemotherapeutic agents such as cytarabine and daunorubicin and HO-1 downregulation favors apoptosis [100]. The same results are observed in chronic myeloid leukemia resistant to imatinib by induction of autophagy [101,102]. Furthermore, in multiple myeloma the high expression of HO-1 stimulates the autocrine production of IL-6, one of the most important survival factors, and increases drug resistance and disease [103]. 

However, in some types of tumor, the role of HO-1 is controversial. Recently, it has been demonstrated that stable HO-1 overexpression retards hepatocellular carcinoma (HCC) progression, downregulating several onco-microRNA among which the most stably downregulated are miR-30d and miR-107 [104]. Moreover, in lung mucoepidermoid carcinoma, a rare subtype of non-small-cell lung cancer, HO-1 inhibits tumor growth by downregulating the oncogenic miR-378 and matrix metalloproteinases and this is confirmed in a murine xenograft model [105]. This different action of HO-1 overexpression in tumor is also related to invasiveness and metastatic potential, as described in paragraph 3. 

## 3. HO-1 in Invasiveness, Angiogenesis, and Metastatic Potential

The gain of a metastatic phenotype is a key step in cancer progression and involves the acquisition of even more aggressive features that increase cell ability to move, overrun surrounding tissue, and favor the growth of new blood vessels in order to increase tumor mass and reach the bloodstream. Different findings prove the involvement of HO-1 in all these aspects of cancer progression.

HO-1 overexpression increases viability, proliferation, and angiogenetic potential of melanoma cells favoring metastasis formation and decreasing tumor-bearing mouse survival [106]. Similarly, the high expression of HO-1 is associated with tumor invasiveness and poor clinical outcome in non-small cell lung cancer patients [107].

Moreover, HO-1 overexpression positively correlates to the thymidine phosphorylase (TP), a proangiogenic enzyme, overexpressed in different human tumors such as NSCLC, breast, and colorectal cancers [108,109] and contributes to higher microvessel density, metastasis, more advanced tumor stage, and poor prognosis [109]. HO-1 upregulation is also involved in osteoponin-induced glioma cell invasion and migration [110] and in breast cancer proliferation [111]. Moreover, it has been shown that brain-derived neurotrophic factor (BDNF), a potent neurotrophic factor involved in cancer cell metastasis and migration, increases the migration of colon cancer cells by regulating VEGF/HO-1 pathway [112].

In NSCLC, the invasive and migratory abilities induced by the exposure to high concentrations of glucose [113] significantly increase after HO-1 overexpression and decrease after HO-1 silencing, being strictly correlated with the expression level of metastasis-associated proteins EGFR, CD147, and MMP-9 [70]. Moreover, in the same kind of tumor cells, resveratrol inhibits metastasis formation, downregulating the expression of HO-1 and the level of matrix metalloproteinases [114]. 

It is noteworthy that HO-1 upregulation has been associated with tumor cell protection against anoikis, a specialized type of apoptosis which follows matrix detachments, contributing to metastatic properties of human fibrosarcoma cells [115]. 

Furthermore, the epithelial-to-mesenchymal transition (EMT) plays a key role in metastatic process [116,117]. It has been shown that in colon cancer cells the glucose-regulated protein 78 (GRP78) favors migration ability and invasiveness through the induction of vimentin expression, the reduction of E-cadherin level, and the activation of Nrf2/HO-1 signal pathway [118]. Moreover, in ovarian cancer cells, it has been demonstrated that the inhibition of autophagy promotes EMT through the increase in intracellular ROS and in the expression of HO-1 and that HO-1 inhibition treatment impairs the migration and invasion by reversing EMT [119].

Tumor invasiveness and metastasis are strictly related to the stimulation of angiogenesis. The role of HO-1 in angiogenesis is well documented both in vitro and in vivo [6,14,67,120,121,122]. HO-1 derived CO stimulates blood vessel formation [14] and induces VEGF [123] and stromal-cell-derived factor 1 (SDF-1) leading to angiogenesis [124]. Moreover, HO-1 overexpression promotes angiogenesis in urothelial carcinoma cells [121] and in human pancreatic cancer [125]. In colorectal cancer cells, HO-1 inhibition suppresses the expression of HIF-1α and VEGF and decreases the degree of angiogenesis in a mouse xenograft model [126]. In tumor-bearing mice, the use of ZnPPIX to inhibit HO-1 prevents peritoneal metastasis of gastric cancer by reducing angiogenesis [127]. In addition, as recently reported by Lin and coworkers, HO-1 expression in the tumor niche promotes lung metastasis by controlling VEGF and IL-10 production [122].

As previously described in paragraph 2, the role of HO-1 in tumor biology is controversial and specifically context-dependent. In particular, in some experimental models, the increased HO-1 expression is associated with the inhibition of angiogenesis, and the slowing of tumor growth. In fact, the upregulation of HO-1 in prostate cancer PC3 cells is associated with a decrease in cell proliferation and invasiveness [128] due to HO-1 dependent downregulation of the proangiogenic mediators VEGF and MMP-9 [129,130]. It has been also demonstrated that in lung mucoepidermoid carcinoma (MEC) the Nrf2-dependent HO-1 activation is associated with a tumor-suppressive phenotype characterized by a strong downregulation of MMP-9 and MMP-13 and the attenuation of the metastatic potential [105]. In addition, carbon monoxide exposure inhibits tumor proliferation and microvascular density in xenotransplanted tumors [131] and human pancreatic cancer cells treated with carbon monoxide-releasing molecules show a significant inhibition of cell proliferation [131]. Finally, Skrzypek and coworkers identified a particular subtype of non-small-cell lung carcinoma (NSCLC) where HO-1 acts as a tumor suppressor, inhibiting cancer cell proliferation, migration, angiogenesis, and tumor growth. Indeed, they demonstrated that the stable overexpression of HO-1 upregulates tumor-suppressive microRNA and downregulates the expression of onco- and angio-microRNA, such as miR-378 [132]. 

## 4. HO-1 in Tumor Immune-Escape

Tumor microenvironment consists of infiltrating immune cells, endothelial cells, extracellular matrix, and signaling molecules. Growth, invasiveness, and metastatic ability of tumors are mediated by cell interactions in this microenvironment [133,134]. Malignant cells have developed different mechanisms to escape from the immune system [135,136]. Many types of leukocytes such as B and T lymphocytes, neutrophilis, macrophages, dendritic (DC), and natural killer (NK) cells can infiltrate tumors [137]. Even though tumor-specific cytotoxic T lymphocytes (TILs) and NK can eliminate tumor cells, there is increasing evidence that, in solid tumors and hematological malignancies, the high number of T regulatory (T_reg_), a specialized sub-population of T cells, acts to suppress the immune response [138,139,140,141].

Numerous reports have shown that the degradation pathway of heme groups is involved in the regulation of the immune system [142] and that HO-1 plays an important role in the modulation of immune reactions within the tumor [143,144], for instance in the recruitment of immune cells [145]. Within the tumors, HO-1 can be expressed both in cancer cells and/or in infiltrating leucocytes [6].

In colorectal cancer cells (CRC), HO-1 expression directly regulates antitumor immunity reducing the expression of ICAM-1 and CXCL10, which in turn inhibits the adhesion and recruitment of T effector (T_eff_) cells, inhibiting cell-mediated cytoxicity [135]. In addition, T_regs_ recruitment is increased in HO-1-dependent manner in 4T1 breast cancer and in tumor-bearing mice [146] and HO-1 expression in cancer cells is related to the increased generation of IL-10 responsible for keeping DC immaturity [147,148].

The main effector of HO-1 in the regulation of the immune system is its metabolic product CO [142,149] which exerts pleiotropic effects in most immune cell types [149]. For instance, CO blocks DC maturation and modulates their cytokine secretion, inducing a tolerogenic phenotype. It also inhibits proliferation and activation of T_eff_, induces T_regs_ expansion [150] and is involved in the early expansion, differentiation, and maturation of myeloid cells into macrophages [142]. Moreover, it has recently been shown that bilirubin can act as endogenous regulator of inflammation, impairing the expression of adhesion molecules [151] and even though the role of HO-1-dependent bilirubin generation in cancer microenvironment has not been demonstrated yet, it seems conceivable that it could play a role in immune-escape. 

As far as HO-1 expression in immune cells is concerned, HO-1 is differentially expressed between CD4^+^CD25^+^ (T_regs_) and CD4^+^CD25^−^ (T_eff_) cell populations [141,143]. In human T_regs_, HO-1 expression directly correlates with the expression of T_reg_-specific marker FoxP3 [152] and HO-1-derived CO seems to be the mediator of the antiproliferative effects on CD4^+^T cells through the block of IL-2 production [153]. Moreover, HO-1 overexpression renders T_regs_ resistant to Fas-mediated apoptosis [154]. Several studies have also demonstrated that T_regs_ play a pathological role in brain tumor progression [155,156,157]. In fact, T_regs_ progressively infiltrate human malignant gliomas and the overexpression of HO-1 is associated with the increased tumor grade and immune suppression [158]. Accordingly, HO-1 expression favors T_regs_ survival to the hypoxic environment and its inhibition with SnPPIX reduces the number of T_regs_, resulting in a survival advantage in glioma bearing-mice [148]. In addition, the upregulation of HO-1 in DC favors T_regs_ recruitment in tumor stromal compartment [159].

However, the presence of T_eff_ remains useful to cancer recognition. Indeed, recently, Longo and coworkers have demonstrated that a fasting-mimicking diet (FMD) regime in combination with a standard chemotherapy, through the downregulation of HO-1 expression in tumor cells, enhances tumor immunogenicity by stimulating the levels of cytotoxic CD8^+ ^tumor-infiltrating lymphocyte and decreasing the levels of T_regs_, leading to a major delay in breast and melanoma progression [146].

Moreover, in the tumor microenvironment, the presence of macrophages dramatically influences cancer progression. Indeed, macrophages with a specific polarization phenotype can differentially modulate the growth and the invasiveness of cancer cells, regulating the immune response of T cells as well as angiogenesis and metastatic growth. Depending on their polarization state, macrophages (M1) are able to kill tumor cells and present tumor-associated antigens or exert tumor supportive activities (M2) by promoting invasion and metastasis [160]. HO-1 expression in stromal macrophages in different cancer tissue (e.g., gliomas and melanomas) was observed in the early 2000s [161,162]. HO-1 is involved in macrophage polarization toward a pro-angiogenic, IL-10 producing, M2 phenotype [163,164,165]. It has been demonstrated that HO-1 positive macrophages induce suppression of the immune response [166] and are associated with poor outcome of cancer patients [160,161,167,168], as shown in breast cancer where HO-1 positive tumor-associated macrophages (TAMs) correlate with an accelerated tumor growth [111]. Moreover, in a transplanted model of pancreatic ductal adenocarcinoma, a subset of M2 macrophages expressing fibroblast activation protein-α (FAP) [169] mediate immune suppression by the induction of HO-1 [166]. 

Furthermore, a key role in tumor recognition is also played by NK cells which are crucially involved in the early immune-response to tumor cells [170]. In fact, activated NK cells express important receptors (NKp30, NKp44, and NKp46) [171] that recognize specific ligands in tumor [172] inducing apoptosis or recruiting other immune cells [173]. Yet, the interplay between HO-1 and NK-mediated tumor recognition is still poorly understood. It has been shown that the inhibition of HO-1 in different cervical cancer cell lines (CCC), increases the expression of INF-γ and TNF-α in co-cultured NK and restores the expression of NKG2D, NKp30, and NKp46, markers of NK activation [174].

## 5. Nuclear Localization of HO-1

As previously described, HO-1 protein can localize in multiple subcellular compartments, including mitochondria and caveolae, and also into the nucleus after its c-terminal cleavage. There are several studies showing that HO-1 is preferentially localized into the nucleus in different tumors such as prostate, lung, and oral cancer as well as in chronic myeloid leukemia [13,175,176,177,178,179] and that this localization correlates with cancer progression [13]. In addition, HO-1 nuclear localization has been observed in metastatic cells of prostate cancer [128] and in head and neck cancer [178] as well as in lung A549 and in prostate DU145 cancer cell lines. Moreover, in lung cancer tissue expressing high levels of HO-1, the signal peptide peptidase (SPP) catalyzes the intramembrane cleavage of HO-1 and positive nuclear staining is also evident [177]. In the same paper, it has been shown that the overexpression of a truncated form of HO-1 in cervical carcinoma and in lung cancers promotes cell proliferation and invasion and the tumorigenic effect has also been demonstrated in a mouse model [177]. Interestingly, in chronic myeloid leukemia, HO-1 nuclear translocation is responsible for imanitib resistance. In fact, the treatment with the protease inhibitor Ed64, able to prevent HO-1 nuclear translocation, enhances imatinib-induced cytotoxicity [179]. 

In support of the hypothesis that nuclear localization of HO-1 could be involved in cancer progression, Biswas and coworkers have shown that nuclear HO-1 interacts with Nrf2, increasing its stabilization. The authors provided evidence of a preferential induction of NQO1 and glucose-6-phosphate dehydrogenase (G6PDH) which favors cell survival by shifting the metabolism to the hexose monophosphate pathway [24,180].

However, it is important to note that there are some opposite observations. In fact, in prostate cancer, nuclear HO-1 localization exerts anti-tumorigenic effects [181] for instance inhibiting cell proliferation, migration, and invasion both in vitro and in vivo [128] or acting on the NF-kB pathway and preventing the angiogenetic switch [129]. Recently, it has been demonstrated that nuclear HO-1 in PCa prostate cancer through the binding to the promoter region of prostate-specific antigen (PSA) acts as a repressor of its transcriptional activity [181]. 

Nevertheless, the mechanism underlying HO-1 nuclear translocation and its pathological significance among the different tumors needs to be investigated further. 

## 6. HO-1 as a Possible Biomarker

Recently, some studies have been carried out to explore the prognostic significance of HO-1 overexpression in human cancers as well as the possible correlation with tumor clinical features and outcome [84,182,183].

NSCLC patients with metastasis and advanced stage disease (III-IV) exhibit a higher and significant expression of HO-1 as compared to early stage and non-metastatic patients and the expression of HO-1 inversely correlates with patient survival [70]. Notably, HO-1 expression together with Nrf2 activation correlate with tumor differentiation, Nevin staging, and metastasis in gallbladder cancer progression and, as far as Nrf2 is concerned, to overall survival [182]. In cholangiocarcinoma, high HO-1 expression in tumor tissue is associated with poor prognosis and lower survival evaluated using the Kaplan–Meier method [184]. 

Both HO-1 and Nrf2 expression are elevated in specimens of bladder cancer [185] and, in particular, in tissue specimens from patients with primary non-muscle-invasive bladder cancer (NMIBC) [186] the high expression of HO-1 was detected in 33% of all primary NMIBC cases examined and the expression was significantly associated with adverse pathological characteristics, tumor progression, lower recurrence-free survival, and progression-free survival [186]. Therefore, these findings suggest that high HO-1 expression may be associated with an increased risk of tumor recurrence and disease progression in primary NMIBC, thus indicating a more aggressive phenotype.

Moreover, in neuroblastoma samples, the univariate Kaplan–Meier analysis revealed that high HO-1 RNA expression levels are associated with an unfavorable prognosis [147] and, in histological samples of human glioma, HO-1 expression was higher in comparison to non-malignant brain tissue [187] with no differences among the various tumor grades. However, HO-1 protein expression was associated with a worse prognosis of grade II and III astrocytoma patients [187].

Furthermore, the relationship between high HO-1 expression and chronic myeloid leukemia (CML) progression and relapse has been analyzed. HO-1 mRNA level in CML patients was significantly higher in comparison to donor samples, and its expression significantly increased in relapsing disease, indicating that HO-1 may be a potential molecular indicator for the progression of disease [188].

## 7. Perspectives and Innovations

In this review, we highlighted the role of HO-1 in favoring tumor progression and eventually its possible use as a biomarker. However, the existence of contrasting data has also been analyzed as it could limit the value of our hypothesis. In this context, we believe it is worth underlining that the effect of HO-1 overexpression is carried out by its metabolic products. Their actions, indeed, can be actually different in the different tissues due to the activity of the other enzymes involved in the degradation pathway of heme. In fact, biliverdin reductase undergoes a tissue- and differentiation-dependent distribution [189,190], influencing the generation of bilirubin. Furthermore, the ability to quench free iron generated from HO-1 can play a role in cancer progression as highlighted by Alkhateeb and Connor [191] who deeply analyzed the role of ferritin expression in cancer cell resistance and in inducing a favorable microenvironment. It is conceivable that the fine balance among all the products derived from heme degradation pathway can lead to pro- or anti-tumorigenic effects.

Finally, it is also important to consider the role of HO-1 in microRNA biogenesis and regulation which are highly tissue- and cell-specific [10] and differently involved in tumor biology. 

It is clear that the specific role of HO-1 in tumor progression is far from being completely understood but it is clear as well that HO-1, and perhaps all the heme degradation pathways, can be strategically modulated in cancer therapy.

So far, different HO-1 inhibitors have been identified [192]. The metalloporphyrins (e.g., Sn-, Zn-, and Cr-protoporphyrin), structurally similar to heme, have been used as prototypic inhibitors of HO-1, even though their application is limited by several pitfalls [10]. In fact, ZnPPIX acts also on other pathways such as indoleamine-2,3-dioxygenases, cyclin D1, and Wnt/β-catenin and, due to its poor solubility, its application in vivo is limited [110]. Thus, several modifications of ZnPPIX have been made. The pegylated form PEG-ZnPPIX is characterized by increased circulation time, enhanced permeability and retention [193]. Also, the styrene maleic acid modification SMA-ZnPPIX shows increased effectiveness in term of solubility and uptake [194]. Recently, other water-soluble imidazole-based molecules have been proven to be HO-1 inhibitors with a better specificity towards HO-1 than protoporphyrin compounds, increasing the chances of developing new molecules [192]. In addition, other therapeutic opportunities can derived from the studies of specific siRNA to silence HO-1 or from new tools able to modulated microRNAs involved in HO-1 activity. Finally, the modulation of biliverdin reductase activity [189] or the downregulation of ferritin levels [191] have been proposed as strategies in cancer therapy as well.

## 8. Conclusions

**Figure 2:**
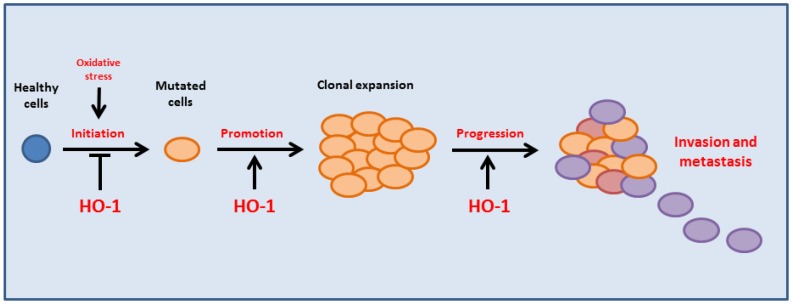
HO-1 induction prevents cell transformation acting through an antioxidant protective mechanism in healthy cells. However, malignant cells can take advantage of HO-1 upregulation favoring tumor growth, invasion, and metastasis.

The upregulation of HO-1 plays a pivotal role in cell adaptation to oxidative/electrophilic stress and to cytotoxic insults. Even though the activation of this powerful system in healthy cells is essential in the prevention of carcinogenesis [69], its activation becomes deleterious in cancer progression. Indeed, a sustained activation of HO-1 in cancer favors tumor growth, metastasis and invasiveness potential, resistance to therapy, as well as modulation of microenvironment and immune-escape, leading to a poor outcome (Figure 2). 

**Figure 3:**
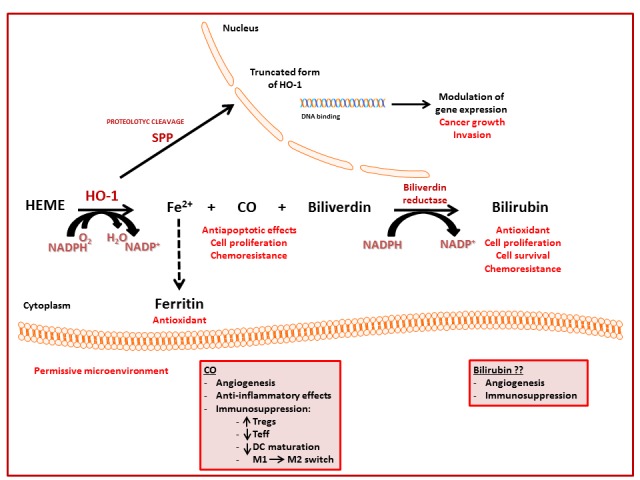
Schematic representation of HO-1 metabolism. HO-1 substrate, co-factors and metabolic products are indicated together with their recognized or hypothesized effects.

The multifaceted role of HO-1 in cancer is mediated mainly by the action of its metabolic products that exert antioxidant, antiapoptotic, and immunomodulatory effects. However, HO-1 could also play a role independently on its enzymatic activity acting as a transcriptional regulator at the nuclear level (Figure 3). In conclusion, the in-depth investigation of these multiple pathways involving HO-1 in cancer biology may improve the therapeutic and the translational potential of HO-1 modulation.
